# Magnesium for the Management of Chronic Noncancer Pain in Adults: Protocol for a Systematic Review

**DOI:** 10.2196/11654

**Published:** 2019-01-11

**Authors:** Rex Park, Anthony M-H Ho, Gisèle Pickering, Lars Arendt-Nielsen, Mohammed Mohiuddin, Ian Gilron

**Affiliations:** 1 School of Medicine Queen's University Kingston, ON Canada; 2 Department of Anesthesiology and Perioperative Medicine Queen's University Kingston, ON Canada; 3 Université Clermont Auvergne Clermont-Ferrand France; 4 Aalborg University Aalborg Denmark; 5 Department of Biomedical and Molecular Sciences Queen's University Kingston, ON Canada; 6 Centre for Neuroscience Studies Queen's University Kingston, ON Canada

**Keywords:** chronic pain, magnesium, noncancer pain, pain management, placebo

## Abstract

**Background:**

Chronic pain is a highly prevalent and complex health problem that is associated with a severe symptom burden, as well as substantial economic and social impact. Many patients with chronic pain still suffer from unrelieved or undertreated pain due to the incomplete efficacy and dose-limiting adverse effects of current therapies. Long-term and high-dose opioid use has considerably increased in the past 20 years despite limited evidence supporting its effectiveness in several chronic pain conditions, and serious concerns have emerged regarding adverse effects and potential misuse. Until recently, the steady increase in opioid prescribing rates has been associated with rising opioid-related mortality and other serious problems, emphasizing the need for better nonopioid therapies. Emerging evidence supports the safe use of magnesium in controlling chronic pain, but its overall efficacy and safety is still unclear.

**Objective:**

This paper aims to assess the efficacy and safety of magnesium compared with a placebo for the treatment of chronic noncancer pain.

**Methods:**

We will conduct a detailed search on Cochrane Central Register of Controlled Trials, MEDLINE, and EMBASE from their inception until the date the searches are run to identify relevant randomized controlled trials. The reference lists of retrieved studies as well as Web-based trial registries will also be searched. We will include randomized double-blind trials comparing magnesium (at any dose, frequency, or route of administration) with placebo using participant-reported pain assessment. Two reviewers will independently evaluate studies for eligibility, extract data, and assess trial quality and potential bias. Risk of bias will be assessed using criteria outlined in the Cochrane Handbook for Systematic Review of Interventions. Primary outcomes for this review will include any validated measure of pain intensity or pain relief. Dichotomous data will be used to calculate the risk ratio and number needed to treat or harm. The quality of evidence will be assessed using the Grading of Recommendations Assessment, Development and Evaluation approach.

**Results:**

This protocol is grant-funded and has undergone a peer-review process through the Queen’s University Department of Anesthesiology and Perioperative Medicine Vandewater Endowed Studentship. This project is also supported, in part, by the Chronic Pain Network of the Canadian Institutes of Health Research Strategy for Patient-Oriented Research. The electronic database search strategies are currently being developed and modified. The entire review is expected to be completed by January 1, 2019.

**Conclusions:**

The completion of this review is expected to identify available high-quality evidence describing the efficacy and safety of magnesium for the treatment of chronic noncancer pain.

**International Registered Report Identifier (IRRID):**

PRR1-10.2196/11654

## Introduction

### Description of the Condition

Chronic pain is a significant health problem given its prevalence, impact on quality of life, economic burden, and difficult management. Chronic pain is defined as pain that persists for over 3 months or past the normal time for tissue healing [1]. However, in most cases the duration of the pain is much longer [2,3]. For example, one Canadian study found that over 65% of people with chronic pain have experienced pain for over 5 years [2]. Chronic pain is associated with increased mortality and has major negative impacts on daily living activities and work-related outcomes, such as employment status, days missed from work, and productivity [4]. Depression and anxiety are highly prevalent in the chronic pain population [5]. Individuals living with chronic pain have double the risk of suicide compared with those who do not [6]. Chronic pain is one of the most common reasons for medical visits and is estimated to affect 1.5 billion people worldwide [7-9]. Approximately 30% of adults in the United States and up to 19% of adults in Canada experience chronic pain [10,11]. The direct health care and productivity costs of chronic pain are as high as US $635 billion per year in the United States and Can $43 billion per year in Canada, which exceed the annual costs from cancer and heart disease [11,12]. Pharmacological agents such as antidepressants, anticonvulsants, opioids, nonsteroidal inflammatory drugs, and muscle relaxants are frequently used for pain management [13,14]. Despite the variety of treatments available, they have limited efficacy and dose-limiting adverse effects, leaving a significant unmet need for sufferers [13]. Increases in opioid prescriptions for chronic pain have been associated with increases in opioid-related mortality due to accidental overdose and in the number of individuals requiring treatment for opioid-misuse disorders [15].

### Description of the Intervention

Magnesium is the fourth most abundant cation in the human body and plays a fundamental role in a variety of physiological processes [16,17]. Magnesium serves as a cofactor in over 300 enzyme systems necessary for regulating blood pressure, protein synthesis, muscle contraction, and blood glucose [18,19]. In the nervous system, magnesium is important for neurotransmission and neuromuscular coordination [20]. There is evidence supporting the use of magnesium supplementation in a variety of health problems, including preeclampsia or eclampsia, cardiac arrhythmias, migraine headaches, metabolic syndrome, diabetes, hyperlipidemia, asthma, and premenstrual syndrome [19,20]. Treatment with magnesium is inexpensive and has relatively few, usually mild, side effects [17,21]. Magnesium supplementation can be given orally or parenterally and is available in a variety of formulations [17]. This review will consider all magnesium formulations and dosing regimens when used in the treatment of chronic pain.

### How the Intervention Might Work

*N-* methyl-d-aspartate (NMDA) receptors are active contributors to pain transmission [22,23]. NMDA receptors are found on postsynaptic spinal neurons in the dorsal horn of the spinal cord [24]. Under normal conditions, the NMDA receptor ion channel is blocked by magnesium ions found in nervous tissues [20,25]. However, if there is sustained depolarization of the postsynaptic membrane, such as that from high-frequency pain stimulation or nerve trauma causing abnormal impulse propagation toward the spinal cord, the magnesium plug is removed and calcium enters the cell [13,24]. Increases in intracellular calcium lead to an increase in the intensity of pain through a process termed wind-up [13,24]. In wind-up, the spinal dorsal horn neurons have an amplified and prolonged response to subsequent inputs [23,24]. The influx of calcium can also activate various effector molecules and cause downstream changes [24]. These effector molecules can promote mechanisms of synaptic plasticity, such as long-term potentiation, which can result in elevated sensitivity and activity of dorsal horn neurons [24]. This phenomenon is known as central sensitization and manifests as a heightened response to noxious (hyperalgesia) and normally nonnoxious (allodynia) stimuli [22,24]. Both wind-up and central sensitization are plausible mechanisms for chronic pain states [13]. Magnesium administration modulates NMDA receptor-driven activity by acting as a physiological blocker of the NMDA receptor ion channel [16,26-28]. It is expected that through this mechanism, magnesium administration may prevent wind-up and central sensitization and dampen the activity of the dorsal horn neurons, ultimately reducing the pain experience.

### Why It Is Important to Do This Review

Chronic pain is a common and complex health problem that has a marked negative impact on patients’ quality of life, physical and mental health, family relationships, employment, and economic well-being [29,30]. In North America, clinicians have increasingly prescribed opioids for chronic pain in efforts to improve pain management, despite the relatively modest evidence base and lack of rigorous research demonstrating its long-term effectiveness [31-33]. The high prescribing rates have been accompanied by significant consequences. Deaths from prescription opioid overdoses quadrupled in the last 15 years in the United States, with >200,000 prescription opioid-related deaths since 1999 [31,34]. Although the opioid crisis has been receiving heavy attention from the government and regulatory bodies, which resulted in a decrease in opioid prescriptions since 2013, the prescribing rates still remain very high in various areas across North America [34]. Additionally, continuing efforts to curb the opioid crisis is unlikely to be an effective long-term solution to the chronic pain crisis [35]. The nature of attention given to the opioid crisis may worsen the stigma associated with proper use of prescription opioids, as well as the stigma of those who are successfully finding pain relief from appropriate prescription opioid use, and result in patients being aggressively tapered off prescription opioids without other nonopioid strategies to help control their pain [35,36]. Therefore, the chronic pain crisis should be viewed and dealt with in a larger context in addition to the opioid crisis. This may involve improving public education on chronic pain, reducing stigma of those living with chronic pain, and increasing accessibility of services [35]. Due to the side effects and uncertain benefits associated with opioid use, alongside the limited accessibility of nonopioid therapies, our review will specifically focus on better understanding chronic pain clinically in terms of what nonopioid strategies are effective in the management of chronic pain.

However, finding an alternative treatment for chronic pain remains a major challenge. Many patients with chronic pain are still in pain despite treatment [37]. Growth in chronic pain research in recent decades has led to the advent of some novel agents, but we have yet to address the problem fully [13,37]. Emerging evidence supports the safe use of magnesium in controlling chronic pain, but there is no consensus regarding its clinical effects [17,38]. The findings of this systematic review will help elucidate magnesium’s efficacy and safety as an alternative for controlling pain. This study seeks to improve the quality of life of patients with chronic pain and inform future management strategies for chronic pain.

### Objectives

This paper aims to assess the efficacy and safety of magnesium compared with placebo for the treatment of chronic noncancer pain.

## Methods

We have prepared this protocol in accordance with the Preferred Reporting Items for Systematic Reviews and Meta-Analyses (PRISMA) Protocol guidelines [39]. The systematic review will also be carried out in accordance with recommendations specified in the PRISMA statement [40].

### Criteria for Considering Studies for This Review

#### Types of Studies

The review will include randomized, double-blind, placebo-controlled trials that evaluate the efficacy or safety of magnesium in the treatment of chronic noncancer pain. We will exclude studies that are nonrandomized or nonblinded, studies of experimental pain, case reports, and clinical observations.

#### Types of Participants

We will include studies with adults aged 18 years and over reporting any type of chronic noncancer pain for at least 3 months (12 weeks). Chronic noncancer pain can include persistent (eg, chronic low back pain, fibromyalgia) and intermittent (eg, migraine) pain. Patients with terminal cancer or other terminal illnesses will be excluded.

#### Types of Interventions

We will focus on magnesium at any dose or frequency, by any route, administered for the relief of chronic pain.

#### Comparison

The intervention will be compared to placebo. Studies with other active controls will be included only if they also have a placebo control.

#### Types of Outcome Measures

We will assess participant-reported measures of pain intensity or pain relief using validated methods.

#### Primary Outcomes

The primary outcomes for this review will include any validated measure of pain intensity or pain relief. We will focus on Initiative on Methods, Measurement, and Pain Assessment in Clinical Trials definitions for benefit in chronic pain studies [41].

#### Secondary Outcomes

Secondary outcomes include (1) any pain-related outcome indicating some improvement (eg, improved function); (2) withdrawals due to lack of efficacy, adverse events, and for any cause; (3) participants experiencing any adverse event; (4) participants experiencing any serious adverse event; and (5) specific adverse events (eg, sedation).

### Search Methods for Identification of Studies

#### Electronic Searches

We will conduct a detailed search on Cochrane Central Register of Controlled Trials, MEDLINE, and EMBASE from their inception until the date the searches are run. The search will be limited to studies published in English. The search will include terms relating to the magnesium and chronic noncancer pain. The search strategy for Ovid MEDLINE was developed in consultation with a librarian with expertise in literature searches (Multimedia Appendix 1).

#### Searching Other Resources

We will also review the bibliographies of any randomized controlled trials identified for relevance, as well as search clinical trial databases (ClinicalTrials.gov) and the World Health Organization International Clinical Trials Registry Platform to identify additional published or unpublished data.

### Data Collection and Analysis

#### Selection of Studies

We will export search results to EndNote and remove duplicates. Two reviewers will independently evaluate studies for eligibility. Screening will be performed on titles and abstracts, and full text screening will be performed on citations felt to be potentially eligible. We will exclude studies that clearly do not satisfy the inclusion criteria. Disagreements between the reviewers will be resolved by discussion and consensus. If necessary, a third reviewer will be consulted. The screening and selection process will be presented using a PRISMA flowchart and reasons for exclusion will be reported.

#### Data Extraction and Management

Data from selected studies will be independently extracted by 2 reviewers using standardized data extraction forms. The forms will capture information about the chronic pain condition; number of participants treated; participant characteristics; inclusion and exclusion criteria; type of magnesium used; magnesium levels before and throughout the treatment period; other study drugs used; dose, frequency, and route of administration of magnesium and other study drugs; study duration and follow-up; study design; primary and secondary outcome measures; and results.

#### Assessment of Risk of Bias in Included Studies

Risk of bias for each study will be independently assessed by 2 reviewers using criteria outlined in the Cochrane Handbook for Systematic Review of Interventions [42]. Disagreements between reviewers will be resolved with discussion and consensus. If necessary, a third reviewer will be consulted.

#### Measures of Treatment Effect

We will use dichotomous data to calculate the risk ratio with 95% CIs. A fixed-effect model will be used unless significant statistical heterogeneity is found.

We will calculate the number needed to treat by taking the reciprocal of the absolute risk reduction. We will calculate the number needed to harm in the same manner for unwanted effects.

#### Dealing With Missing Data

For missing data, we will utilize the intention-to-treat analysis. The intention-to-treat population will include participants who were randomized, received at least 1 dose of the assigned study intervention, and provided at least 1 postbaseline assessment. Missing participants will be assigned zero improvement.

#### Assessment of Heterogeneity

Only studies evaluating similar conditions will be combined for analysis to avoid clinical heterogeneity. Clinical heterogeneity will also be assessed visually and by using the I^2^ statistic. When the I^2^ value is higher than 50%, we will consider possible explanations for this.

#### Assessment of Reporting Bias

This review will extract dichotomous data and will not depend on what the authors of the original studies chose to report or not.

We will assess for publication bias using a method that looks for the amount of unpublished data with a null effect needed to make any result clinically irrelevant (usually taken to mean a number needed to treat of ≥10) [43].

#### Data Synthesis and Analysis of Outcomes

Extracted data will be compiled in Microsoft Excel for analysis. Analysis will be carried out using Review Manager (RevMan), Version 5.3 (Copenhagen: The Nordic Cochrane Centre, The Cochrane Collaboration, 2014).

We plan to use a fixed-effect model for meta-analysis. We will use a random-effects model for meta-analysis if it is deemed appropriate to combine heterogeneous studies.

#### Quality of Evidence

The quality of evidence will be rated using the Grading of Recommendations Assessment, Development and Evaluation approach through a ‘summary of findings’ table.

#### Subgroup Analysis and Investigation of Heterogeneity

If sufficient data are available, we plan on conducting a subgroup analysis according to the dose of magnesium, duration of study, different types of pain conditions, and quality of included studies.

## Results

This protocol is grant-funded and has undergone a peer-review process through the Queen’s University Department of Anesthesiology and Perioperative Medicine Vandewater Endowed Studentship. This project is also supported, in part, by the Chronic Pain Network of the Canadian Institutes of Health Research Strategy for Patient-Oriented Research. The protocol was submitted to PROSPERO on July 4, 2018 and is currently being assessed. The electronic database search strategies are currently being developed and modified. The entire review is expected to be completed by January 1, 2019.

## Discussion

Management of chronic pain is ineffective in many individuals, and the increased prescribing of opioids for alleviating chronic pain has been associated with significant consequences. In attempts to curb the opioid crisis, some patients who appropriately use prescription opioids and find relief are being tapered off this treatment and left with limited access to other nonopioid therapies. This review aims to address these issues by better understanding chronic pain clinically, and investigating whether magnesium can serve as a safe alternative for patients who find limited relief from current therapies or have been aggressively tapered off opioids. The completion of this review is expected to identify available high-quality evidence describing the efficacy and safety of magnesium for the treatment of chronic noncancer pain. The results of this review may guide future research in this area and contribute to the development of evidence-based treatment guidelines for the management of chronic noncancer pain.

## Appendix

Multimedia Appendix 1 Search strategy developed for Ovid MEDLINE.

Multimedia Appendix 2 Peer-reviewer report from the Queen’s University Department of Anesthesiology and Perioperative Medicine Vandewater Endowed Studentship.

## Abbreviations

NMDA: N-methyl-d-aspartate
PRISMA: Preferred Reporting Items for Systematic Reviews and Meta-Analyses
