# Supplementary material for: Magnesium for the Management of Chronic Noncancer Pain in Adults: Protocol for a Systematic Review
Source: JMIR Res Protoc. 2019 Jan 11;8(1):e11654. doi: 10.2196/11654 (PMC6330196; doi:10.2196/11654)
Supplement: Multimedia Appendix 1 [file resprot_v8i1e11654_app1.pdf]

1 chronic pain.mp. or Chronic Pain/ (36031)
2 exp Pain/ (360589)
3 peripheral neuropathy.mp. or exp Peripheral Nervous System Diseases/ (144442)
4 neuropathic pain.mp. (17230)
5 Fibromyalgia/ (7699)
6 myofascial pain syndromes/ or temporomandibular joint dysfunction syndrome/ (6265)
7 rheumatic polymyalgia.mp. (71)
8 (chronic adj4 pain).mp. [mp=title, abstract, original title, name of substance word, subject heading word, floating sub-heading word, keyword heading word, protocol supplementary concept word, rare disease supplementary concept word, unique identifier, synonyms] (58478)
9 ((pain* or discomfort*) adj10 (central or complex or rheumat* or muscl* or muscul* or myofasci* or nerv* or neuralg* or neuropath*)).mp. [mp=title, abstract, original title, name of substance word, subject heading word, floating sub-heading word, keyword heading word, protocol supplementary concept word, rare disease supplementary concept word, unique identifier, synonyms] (92071)
10 exp Somatosensory Disorders/ (19931)
11 (fibromyalgi* or fibrosti* or FM or FMS).mp. [mp=title, abstract, original title, name of substance word, subject heading word, floating sub-heading word, keyword heading word, protocol supplementary concept word, rare disease supplementary concept word, unique identifier, synonyms] (30138)
12 ((neur* or nerv*) adj6 (compress* or damag*)).mp. [mp=title, abstract, original title, name of substance word, subject heading word, floating sub-heading word, keyword heading word, protocol supplementary concept word, rare disease supplementary concept word, unique identifier, synonyms] (63883)
13 exp Migraine Disorders/ (25023)
14 1 or 2 or 3 or 4 or 5 or 6 or 7 or 8 or 9 or 10 or 11 or 12 or 13 (630590)
15 MAGNESIUM/ or magnesium.mp. (101935)
16 magnesium sulfate.mp. or Magnesium Sulfate/ (6345)
17 magnesium chloride.mp. or Magnesium Chloride/ (3608)
18 15 or 16 or 17 (101935)
19 14 and 18 (1212)
20 limit 19 to randomized controlled trial (195)
