# Supplementary material for: Magnesium for the Management of Chronic Noncancer Pain in Adults: Protocol for a Systematic Review
Source: JMIR Res Protoc. 2019 Jan 11;8(1):e11654. doi: 10.2196/11654 (PMC6330196; doi:10.2196/11654)
Supplement: Multimedia Appendix 2 [file resprot_v8i1e11654_app2.pdf]

Dr. L. Wang (MD, FRCPC, MSc)
DEPARTMENT OF ANESTHESIOLOGY & PERIOPERATIVE MEDICINE
Queen's University, Faculty of Health Sciences,
Kingston, Ontario K7L 2V7
Kingston General Hospital
76 Stuart Street
Tel: 613 548-7827
Fax: 613 548-1375

May 3, 2018

Dr. I. Gilron
Department of Anesthesiology & Perioperative Medicine
Queen's University & Kingston General Hospital,
Kingston, Ontario K7L 2V7

Dear Dr. Gilron, Ian

Your recent submission to the Department of Anesthesiology and Perioperative Medicine Vandewater Endowed Studentship for a summer student (Rex Park) underwent a peer review process during which it was independently reviewed and scored by at least 2 members of the Department of Anesthesiology.

I am pleased to inform you that your application entitled **Systematic review of magnesium for the treatment of chronic non-cancer pain** was recommended for funding in the amount of $3,000 for which you have agreed to match an additional $3,000. Dana Thompson-Green can assist you with the set-up of your research account which is required before funds can be transferred. We will require a progress report (with financials) to be submitted upon project completion.

Congratulations on your successful application!

Best Regards,

Louie Wang, Research Director
Department of Anesthesiology &
Perioperative Medicine Research Committee

Overall average score= 8/10

Strengths:
Project seems methodologically sound with obvious experienced supervisor. It's good to see that Dr. Ho has been added to this project given his active interest in Mg^{2+}, strong research track record, and enthusiasm for research. It will be good for Rex Park to have interactions with Dr. Ho since it is rare to see a busy clinician with such limited academic time being so active in research and fully enjoying it.

Suggestions:
Would seek some proof of extent of content, preliminary structured search ensure enough content to build meaningful systematic review.
